# Glucose-6-phosphate dehydrogenase deficiency prevalence and genetic variants in malaria endemic areas of Colombia

**DOI:** 10.1186/s12936-016-1343-1

**Published:** 2016-05-26

**Authors:** Sócrates Herrera Valencia, Iván Darío Ocampo, María Isabel Arce-Plata, Judith Recht, Myriam Arévalo-Herrera

**Affiliations:** Caucaseco Scientific Research Center/Malaria Vaccine and Drug Development Center, Carrera 37 2B No. 5 E-08, Edificio de profesionales Bambú, Cali, Colombia; Centro Latino Americano de Investigación en Malaria (CLAIM), Cali, Colombia; Facultad de Salud, Universidad del Valle, Cali, Colombia

**Keywords:** Malaria, G6PD deficiency, Primaquine, Mass drug administration

## Abstract

**Background:**

Glucose 6-phosphate dehydrogenase (G6PD) is an enzyme involved in prevention of cellular oxidative damage, particularly protecting erythrocytes from haemolysis. An estimated 400 million people present variable degrees of inherited G6PD deficiency (G6PDd) which puts them at risk for developing haemolysis triggered by several risk factors including multiple drugs and certain foods. Primaquine (PQ) is a widely used anti-malarial drug that can trigger haemolysis in individuals with G6PDd. Intensification of malaria control programmes worldwide and particularly malaria elimination planning in some regions recommend a more extensive use of PQ and related drugs in populations with different G6PDd prevalence. This a preliminary study to assess the prevalence of G6PDd in representative malaria endemic areas of Colombia by measuring G6PD phonotype and genotypes.

**Methods:**

Volunteers (n = 426) from four malaria endemic areas in Colombia (Buenaventura, Tumaco, Tierralta and Quibdo) were enrolled. Blood samples were drawn to evaluate G6PD enzymatic activity by using a quantitative G6PD test and a subset of samples was analysed by PCR–RFLP to determine the frequency of the three most common G6PD genotypic variants: A−, A+ and Mediterranean.

**Results:**

A total of 28 individuals (6.56 %) displayed either severe or intermediate G6PDd. The highest prevalence (3.51 %) was in Buenaventura, whereas G6PDd prevalence was lower (<1 %) in Tierralta and Quibdo. G6PD A alleles were the most frequent (15.23 %) particularly in Buenaventura and Tumaco. Overall, a high frequency of G6PD A− genotype, followed by A+ genotype was found in the analysed population.

**Conclusions:**

G6PDd based on enzymatic activity as well as G6PD A allelic variants were found in malaria-endemic populations on the Pacific coast of Colombia, where most of malaria cases are caused by *Plasmodium vivax* infections. These infections are treated for 14 days with PQ, however there are no official reports of PQ-induced haemolytic crises. Further assessment of G6PDd prevalence in malaria endemic areas in Colombia is crucial in view of possible mass drug administration for malaria elimination in these regions, as well as implementation of appropriate G6PDd diagnostic methods.

## Background

Glucose 6-phosphate dehydrogenase (G6PD) is a cytoplasmic enzyme involved in prevention of cellular oxidative damage by stimulation of detoxification of free radicals. It catalyzes the production of nicotinamide adenine dinucleotide phosphate (NADPH), which is necessary for maintenance of reduced levels of glutathione (GSH) important to protect erythrocytes from oxidative damage and to reduce susceptibility to haemolysis [1, 2]. However, globally more than 400 million people are estimated to suffer variable degrees of inherited G6PD deficiency (G6PDd). G6PD deficient individuals, many of whom live in malaria endemic regions, are at risk of developing haemolytic crises triggered by several risk factors including multiple drugs and specific foods [3, 4].

The *G6PD* locus is located on chromosome X (Xq28) and displays a great polymorphism with ~190 mutations identified coding for ~400 biochemical or allelic enzyme variants [5–7], some of which lead to functional deficiencies (G6PDd) that are transmitted as X-linked traits [8–10]. This transmission pattern results in G6PDd hemizygous males or homozygous females, whereas heterozygous females can be either normal or deficient in G6PD activity because of mosaicism [2].

Different prevalence of G6PDd has been reported in Africa (20 %), the Mediterranean (4–30 %) and Southeast Asia (10–20 %) [11]. In Latin America, where it has been less studied, prevalence ranges from <2 % in countries such as Guatemala, Mexico and Peru to 16 % in Honduras [2, 12], including Venezuela with a prevalence of ~4 % [13]. The G6PDd African variants or “A” variants are among the most frequent worldwide, and the most common variants reported in the Americas. The A+ variant is characterized by one mutation from Adenine to Guanine at position 376 in exon IV (376 A>G), exhibiting normal to very-mild deficiency (Class IV) whereas the A− variant (Class III) is the result from mutations in two positions in different exons: 376 A>G (exon IV) and 202 G>A (exon V); it presents 8–20 % of normal enzymatic activity. The Mediterranean variant displays mutations in two positions in different exons including the A+ mutation: 376 A>G (exon IV). Class II variant is produced by a change at position 563 in exon VI, corresponding to a transition from cytosine to thymine (563 C>T); it is considered a more severe variant, presenting with <5 % of normal activity [14–16].

Primaquine (PQ), an 8-aminoquinoline, is currently considered the most effective drug to prevent *Plasmodium vivax* clinical relapses [17], as well as in *Plasmodium falciparum* infections to prevent gametocyte development and further parasite transmission to mosquitoes [18]. However, PQ use has been restricted because it can trigger variable degrees of haemolysis in individuals with G6PDd, depending on the variant type. Due to intensification of malaria control programmes worldwide [19, 20] and the perspectives for malaria elimination in some regions where PQ could be massively deployed, it is important to better characterize prevalence of G6PD in malaria endemic communities in these areas. In Colombia, previous studies have indicated a variable G6PDd prevalence in certain regions according to phenotypic analyses: 12 and 12.7 % in Afro-descendants in Buenaventura and Cali, respectively, 3.1 % in a mestizo population in Bogota, 2.2 % in mestizo, Amerindians and Afro-descendants in Medellin, and 14.8 % in healthy individuals and 9.5 % in malaria infected individuals in Turbo [21–25]. However, there is a lack of reports on haemolysis associated to PQ treatment in Colombia, even though ~70 % of the malaria cases are caused by *P. vivax* and treated with PQ. According to the Colombian guidelines for *P. vivax* malaria treatment, PQ must be administered for 14 days at a daily dose of 15 mg/day in combination with chloroquine (CQ) at a total dose of 25 mg/kg; no PQ is currently used for treatment of *P. falciparum* infections [26]. This study aimed to determine the prevalence of both phenotypic and genotypic G6PDd in representative malaria endemic areas of Colombia located on the Pacific coast where *P. vivax* malaria is highly prevalent in endemic communities, many of which are of African descent. This is the first study including genotyping to evaluate locally important G6PDd variants in a large number of individuals from rural malaria endemic areas in the northwest of Colombia. Preliminary evaluation of both G6PD enzyme activity along with variant identification was important to study correlations between genotype and phenotype in these populations.

## Methods

### Ethics statement

Human blood samples were collected as part of a malaria epidemiology study in the context of an International Center of Excellence for Malaria Research (ICEMR) programme. The protocol was reviewed and approved by the IRB of Malaria Vaccine and Drug Development Center-MVDC (Cali-Colombia) (Code 004-2010). The screening process was considered to be of minimal risk according to resolution 8430 of 1993 of the Colombian Ministry of Health (MoH) [27]. Research was all performed in agreement with the Declaration of Helsinki. Adults were asked to sign an informed consent (IC) form prior to inclusion, whereas children seven to 18 years old were asked to provide written informed assent (IA). For children <7 years old only the legal tutor was required to sign the IC for inclusion. Ethical clearance to draw blood samples was obtained by prior written IC, which was previously approved by the Ethical Committee of the MVDC IRB (CECIV) [28].

### Study sites and population

This study was performed in four municipalities: Buenaventura (Valle del Cauca), Quibdo (Choco), Tierralta (department of Cordoba) and Tumaco (Nariño). These municipalities were selected based on the source of the malaria cases reported in the Colombian National Surveillance System (SIVIGILA).

Buenaventura is a municipality located to the West of Colombia on the Pacific coast at 7 m above sea level (m a s l). It has a population of ~392,000 people (~85 % Afrocolombian, ~10 % mestizos) [28]. A total of 334 malaria cases (~71 % *P. vivax*) were reported by SIVIGILA in 2014 [29], placing Buenaventura as a low risk zone (API = 0.8 cases/1000 inhabitants).

Quibdo is located to the northwest of Colombia at 45 m a s l. It has ~160,000 inhabitants and most of them are Afrocolombian (~95 %). This municipality shows ~47 % of malaria cases caused by *P. vivax*, contributing 65 % of all malaria cases in Colombia according to SIVIGILA [29].

Tierralta is located to the northwest of Colombia, 51 m a s l and ~50 km from the Colombian Atlantic coastline with a mean annual temperature of 27.3 °C [30]. Its population is an estimated ~97,000 inhabitants (~44 % living in rural areas) with 86 % mestizo, 8 % African-American, and ~2 % of native-American ethnicity [28]. Tierralta reported 844 malaria cases to SIVIGILA in 2014 (~94.4 % *P. vivax*) [29] and is considered a moderate risk zone according to the Annual Parasite Index (API = 8.6 cases/1000 inhabitants).

Tumaco is a municipality located to the southwest of Colombia on the Pacific coast at ~40 km from the border with Ecuador. It has a population of ~195,000 inhabitants [28]. It differs from the aforementioned localities in that *P. falciparum* is by far the most prevalent malaria pathogen. It reported 1309 malaria cases (1.1 % *P. vivax*, 97.6 % *P. falciparum* and 0.3 % mixed infection) in 2014 [29], which places it as a moderate risk zone (API = 6.7 cases/1000 inhabitants).

### Blood samples

Blood samples used here G6PD studies were randomly selected from a larger group of samples collected of healthy male and female donors of any age, randomly selected in cross sectional studies to determine malaria prevalence in sentinel sites of Buenaventura (n = 118), Quibdo (n = 100), Tierralta (n = 119) and Tumaco (n = 89) [28]. Venous blood (4 mL for adults and 3 mL for children <7 years) was collected by venipuncture into Vacutainer™ tubes (Becton-Dickinson) containing EDTA from a total of 426 volunteers who signed an IC or IA. Malaria infection was evaluated by microscopic and molecular examination. Enzyme activity assays for G6PD were performed using whole blood samples, and DNA was used to analyse G6PD allelic variants. After collection, samples were stored refrigerated at ~4 °C and G6PD activity assays were conducted within 7 days of collection.

### Analysis of G6PD enzymatic activity in human blood

The G6PD activity was measured in all 426 volunteers. Initially, the haemoglobin (Hb) level was determined in all samples using 500 µL of whole blood and an automated haematology analyzer (KX-21 N, Sysmex, Roche). The G6PD activity was measured in 10 µL of whole blood samples using the quantitative G6PD kit from Trinity according to the manufacturer’s instructions (Trinity Biotech, Ireland; Cat No 345-B) and was expressed in terms of Hb concentration. Normal, intermediate and deficient controls supplied by manufacturers were included in each determination. Enzyme activity was determined using a spectrophotometer (Biosystems, BTS 350) at 340 nm and it was reported as international units per gram of Hb(U/gHb). The reference values for enzyme activity were: normal 4.6–13.5 U/gHb, intermediate deficiency 2.1–4.5 U/gHb, and severe deficiency <2.1 U/gHb.

### Molecular analysis to detect A+ , A− and Mediterranean variants

According to a previous estimation of 10 % prevalence of G6PDd in Colombia [2, 21], a subset of 123 samples were randomly selected to study the frequency of the three most common G6PD variants using PCR–RFLP. In addition, all samples with detected G6PDd were included in this analysis. First, PCR reactions were performed to amplify exons IV, V and VI of the *G6PD* gene to characterize the genotypic variants A−, A+ and Mediterranean [31, 32]. DNA was extracted from blood samples using the PureLink™ Genomic DNA Mini Kit according to manufacturer’s recommendations (invitrogen™). All PCR reactions were performed to a final volume of 12.5 µL, using the GoTaq^®^ Colorless Master Mix according to manufacturer’s recommendations (Promega), 2 μL of DNA and specific primers for each region. The PCR conditions used to amplify the three fragments were: 94 °C/5 min, 35 cycles of 95 °C/30 s, 62 °C/30 s, and 72 °C/30 s, and final extension of 72 °C/5 min for regions including mutations 376 A>G (90 bp product) and 202 G>A (109 bp product). For the region including the mutation 563C-T (263 bp product) the PCR conditions for amplification were similar except for annealing and extension time which were 45 s. Finally, the PCR products were detected in 3 % agarose gels. The PCR products were used in RFLP with specific restriction enzymes. All restriction reactions were performed according to manufacturer’s recommendations. The mutation 376 A>G (G6PD A+ variant) was identified using the enzyme Fok I (New England Biolabs). The 202 G>A transition (G6PD A− variant) was detected by restriction with NIaIII (New England Biolabs). Finally, MobII (New England Biolabs) was used to detect the 563 C>T mutation associated with the Mediterranean variant. The digested products were analysed through electrophoresis on 3 % agarose gels.

### Statistical analysis

Descriptive measures (mean, median, standard deviation and range) and a Chi squared test were performed to describe and to test the relationship of variables such as age, gender, previous malaria infection, and number of previous malaria episodes, ethnicity and provenance with the presence of G6PDd (Tables 1, 2). A difference was considered significant if p value was <0.05. The EPIDAT (4.1 version) software and R were used to analyse data.

**Table 1:** Demographic characteristics of selected people and frequency of people with previous malaria episodes by G6PDd classification

Area	Ethnicity	n (%)	Severe	Intermediate	Normal	Overproduction
Buenaventura	Afrocolombian	94	2	12	78	2
	Mestizo	4	0	1	3	0
	Other	20	0	0	20	0
	Total	118	2	13	101	2
Quibdo	Afrocolombian	86	0	1	85	0
	Mestizo	4	0	0	4	0
	Other	10	0	1	9	0
	Total	100	0	2	98	0
Tierralta	Afrocolombian	2	0	0	2	0
	Mestizo	94	0	3	88	3
	Other	23	0	1	21	1
	Total	119	0	4	111	4
Tumaco	Afrocolombian	44	2	1	40	1
	Mestizo	11	0	1	10	0
	Other	34	2	1	30	1
	Total	89	4	3	80	2

Previous malaria episodes					
No	147	1	4	139	3
Yes	279	5	18	251	5
Total	426	6	22	390	8

**Table 2:** Prevalence of G6PDd and summary statistics for G6PD enzyme activity and haemoglobin values for gender classification and areas of study

	n	Min		Median		Mean		Max		Deficiency n (%)	Confidence intervals
		G6PD (U/gHb)	Hb (g/dL)	G6PD (U/gHb)	Hb (g/dL)	G6PD (U/gHb)	Hb (g/dL)	G6PD (U/gHb)	Hb (g/dL)		
Gender											
Male	175	1.40	7.10	7.51	12.90	7.72	12.99	23.8	23.00	7 (4.00)	[1.76, 8.39]
Female	251	1.93	7.10	7.94	12.00	7.79	12.18	15.8	19.70	21 (8.37)	[5.38, 12.68]
Area											
Buenaventura	118	1.70	7.10	7.38	12.00	7.34	12.26	23.80	19.70	15 (12.71)	[7.53, 20.41]
Tierralta	119	2.61	9.00	8.42	12.40	8.50	12.67	15.80	23.00	4 (3.36)	[1.08, 8.90]
Tumaco	89	1.40	7.40	8.30	12.70	8.04	12.51	15.50	18.00	7 (7.87)	[3.49, 16.05]
Quibdo	100	4.17	9.00	6.64	12.20	7.11	12.61	12.65	17.70	2 (2.00)	[0.35, 7.74]
Total	426	1.40	7.10	7.76	12.30	7.76	23.00	23.80	23.80	28 (6.56)	[4.49, 9.47]

## Results

**Fig. 1:**
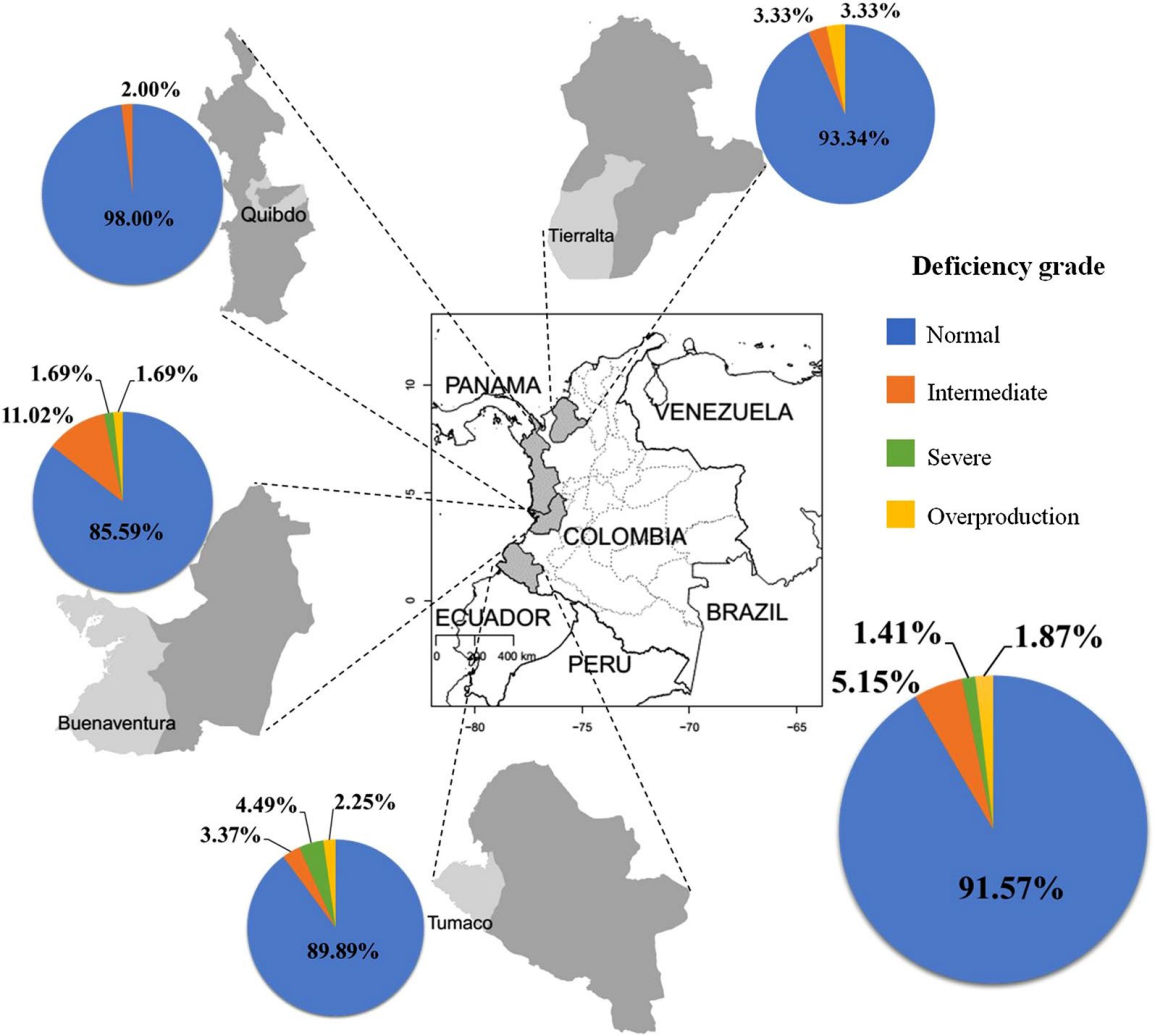
Geographic distribution of G6PD deficiency according to enzyme activity. G6PDd according to levels of enzymatic activity is shown in *pie charts* for each of the four regions in Colombia included in this study, all located on the coast. Average levels including all regions are shown in the *lower right pie chart*

A total of 426 volunteers, 251 females and 175 males, were screened for G6PD activity: 118 from Buenaventura, 100 from Quibdo, 119 from Tierralta and 89 from Tumaco (Table 2). Eleven of the study volunteers presented malaria infection as determined by thick blood smear and PCR [28], but none of them showed G6PDd. No significant differences were found for G6PD activity among males and females. The average G6PD activity was 7.76 U/gHb, with 28 individuals (6.56 %; 7 males and 21 females) showing either severe or intermediate G6PDd reflected in values from 1.40 to 4.45 U/gHb (Fig. 1; Table 2). From these 28 patients, six (1.41 %; 2 females and 4 males) showed severe G6PDd, whereas 22 (5.15 %) showed intermediate deficiency. No differences were found between G6PDd and activity between males and females. There were regional differences in G6PD enzyme activity (p < 0.001) but no significant differences were detected in the prevalence of G6PDd. According to enzyme activity the highest prevalence was found in Buenaventura, where 15 (12.71 %) volunteers showed severe or intermediate deficiency followed by Tumaco, with seven G6PDdcases and the highest number of severe deficiency cases, four with severe deficiency and three with intermediate deficiency. Tierralta and Quibdo, showed the lowest prevalence of G6PDd with only intermediate deficiency (Table 2).

**Fig. 2:**
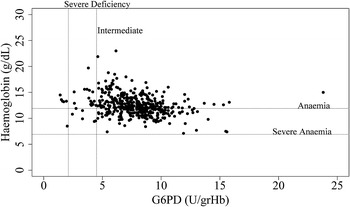
Level of G6PD activity and Haemoglobin. Hb concentration >12 g/dL in females and >13 g/dL in males were considered normal, between 7.0 and 11.9 g/dL mild to moderate anaemia, and <7.0 g/dL corresponded to severely low or severe anaemia

None of the patients with G6PDd had severe anaemia (Hb <7 g/dL) (Table 2 and Fig. 2). Five individuals (four males and one female) with severe G6PDd showed normal levels of Hb (>12 g/dL in females and >13 g/dL in males). However, seven volunteers had mild to moderate anaemia, one female with severe deficiency (7.0–11.9 g/dL in females and 7.0–12.9 g/dL in males), and six volunteers (five females and one male) with intermediate G6PDd (Fig. 2).

**Fig. 3:**
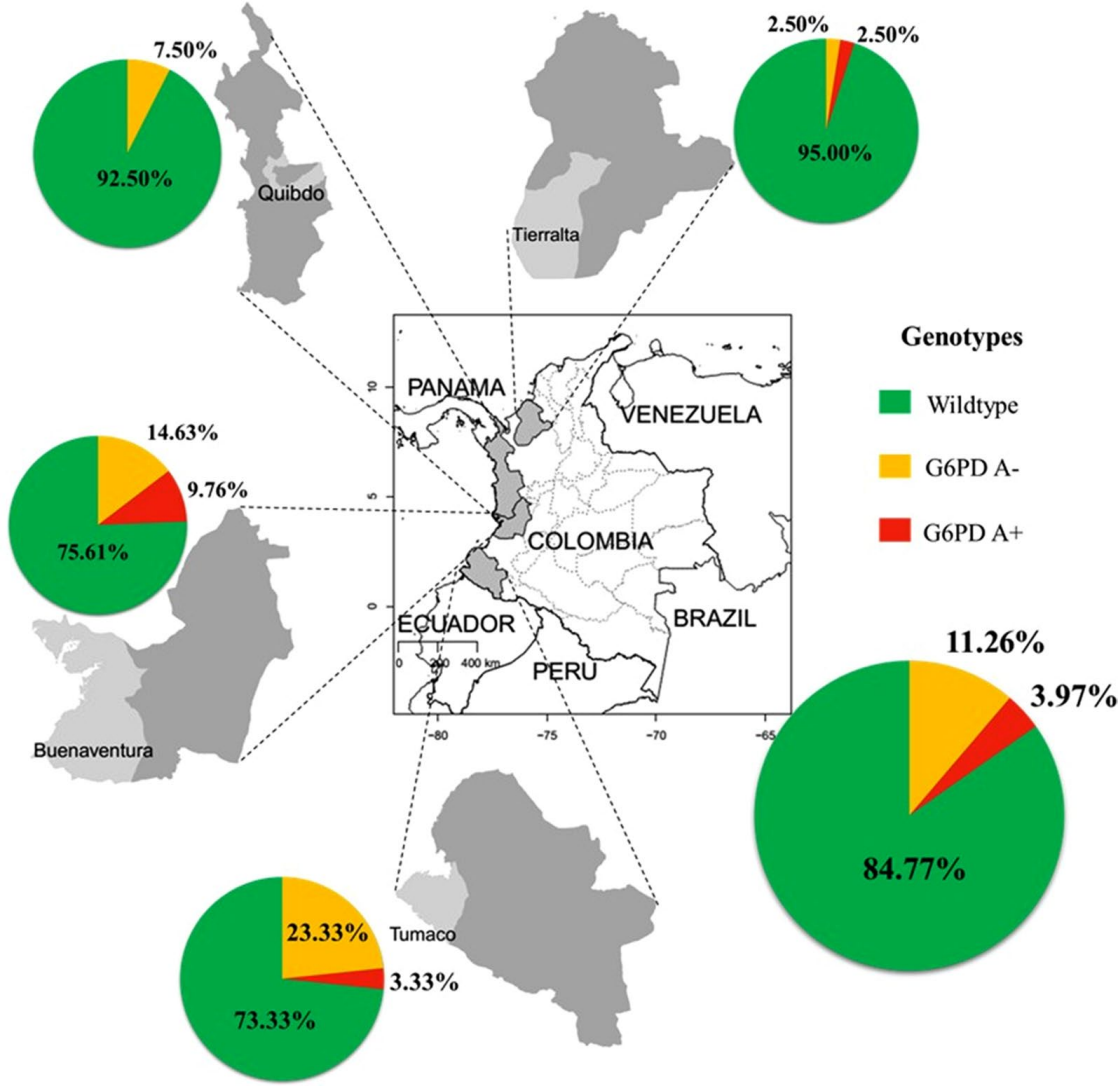
Geographic distribution of G6PD allelic variants identified on the Colombian Pacific coast. G6PD genotypes wild-type, A− and A+ are shown in pie charts for each of the four regions in Colombia included in this study. Average levels including all regions are shown in the *lower right pie chart*

Analysis of G6PD allelic variants by PCR–RFLP was successful for 151 samples, 77 (50.99 %) female and 74 (49.01 %) male individuals, including 123 patients with normal G6PD activity, 22 with intermediate deficiency and six that showed severe deficiency. A total of 23 individuals (15.23 %) showed genotypes associated with G6PD deficiency (Table 3). Only the G6PD genotypes A− or A+ were found. In contrast, no Mediterranean variant was identified. The G6PD A− variant (376 A>G and 202 G>A mutations) was identified in 10 (6.62 %) hemizygous males and seven (4.64 %) females (four heterozygous and three homozygous). The A+ variant, which only includes the 376 A>G mutation, was found in five (3.31 %) hemizygous males, and only one heterozygous female (Table 3). The remaining 128 individuals did not show any of the mutations evaluated in this study. The highest proportion of individuals with G6PD A− and A+ variants was found in Tumaco (26.67 %) and Buenaventura (24.39 %). However, the number of individuals characterized in Tumaco was the lowest among all municipalities (Table 4). The lowest frequency of these two G6PD variants was identified in Tierralta (5.00 %) and Quibdo (7.50 %) (Table 4). The geographic distribution of all variants in this study is summarized in Fig. 3.

**Table 3:** Number of G6PD alleles among individuals according to gender

	Samples n (%)	Genotypes^a^		
		G6PD A− n (%)	G6PD A+ n (%)	Wild type n (%)
Gender				
Male	67 (44.3)	5 (3.31)	4 (2.65)	58 (38.41)
Female	56 (37.09)	6 (3.97)	0 (0.00)	50 (33.11)
Deficiency				
Intermediate	22 (14.57)	2 (1.32)	2 (1.32)	18 (11.92)
Severe	6 (3.97)	4 (2.65)	0 (0.00)	2 (1.32)
Total	151 (100)	17 (11.26)	6 (3.97)	128 (84.77)

^a^The Mediterranean variant, tested by PCR–RFLP, was not found in any of the samples

**Table 4:** Proportion of G6PD genotypes by municipalities

Municipality	Samples (n)	G6PD genotypes^a^	n (%)	Confidence intervals
Buenaventura	41	A−	6 (14.63)	[6.09, 29.86]
		A+	4 (9.76)	[3.17, 24.06]
		Wild type	31 (75.61)	[59.36, 97.09]
Quibdo	40	A−	3 (7.50)	[1.96, 21.48]
		A+	0 (0.00)	[0.00, 10.91]
		Wild type	37 (92.50)	[78.52, 98.04]
Tierralta	40	A−	1 (2.50)	[0.13, 14.73]
		A+	1 (2.50)	[0.13, 14.73]
		Wild type	38 (95.00)	[81.79, 99.13]
Tumaco	30	A−	7 (23.33)	[10.64, 42.70]
		A+	1 (3.33)	[0.17, 19.05]
		Wild type	22 (73.33)	[53.83, 87.02]
Total	151	A−	17 (11.26)	[6.89, 17.67]
		A+	6 (3.97)	[1.63, 8.83]
		Wild type	128 (84.77)	[77.81, 89.90]

^a^The Mediterranean variant, tested by PCR–RFLP, was not found in any of the samples

The enzymatic activity and molecular characterization were compared to evaluate the association between G6PD variants and enzyme deficiency. Eight of 28 individuals with enzymatic deficiency showed one of the G6PD allelic variants analysed in this study, whereas the remaining 20 individuals were wild type for the three variants (Table 3). Two individuals from Buenaventura with intermediate enzymatic deficiency showed the G6PD A+ and G6PD A− variants respectively. Additionally, in this same municipality, one male with the A− variant showed severe deficiency (Table 4). Similarly, in Tierralta two individuals with A+ and A− variants showed intermediate G6PDd (Table 4). Finally, three individuals with the G6PD A− variant in Tumaco showed severe deficiency (Table 4). In this study, the G6PD A− allelic variant was found associated with both severe and intermediate deficiency, whereas the A+ genotype was only associated with intermediate deficiency (Table 4). The remaining 15 individuals with G6PD A allelic variants showed normal activity.

## Discussion

This is the first study performed to evaluate the prevalence of G6PDd in a large number of individuals through enzymatic activity accompanied by genotyping analysis in malaria endemic regions on the Pacific coast in Colombia. This approach allowed to preliminarily evaluate the frequency of allelic variants associated with G6PDd in municipalities from this region of Colombia.

This study found 6.56 % of analysed individuals showing G6PD deficient activity including both intermediate and severe deficiency. This overall frequency is lower than the 10 % prevalence reported previously for Colombia in a recent review which includes studies from Latin America [2, 21], the 12 % reported in Buenaventura for 242 individuals [21], 14.7 % in 508 individuals in Turbo [25] and the 12.7 % found in Cali [23]. However, the results found in this study were higher than previous reports including a G6PDd prevalence of 3.1 % in a mestizo population in Bogotá [22] and 2.2 % in 500 Amerindians and Afro-Amerindians individuals in Medellin [25]. The differences in G6PD activity found in various Colombian studies are probably due to composition of the different populations evaluated; a higher G6PDd prevalence in Afro-descendants is likely, particularly in malaria endemic regions.

The A− and A+ allelic variants are the most often reported globally associated with G6PDd, although the A− variant is the one most frequently evaluated in G6PDd studies in populations of African descent. In this study, 15.23 % of the individuals had G6PD A genotypes, mostly A− (11.26 % A− and 3.97 % A+), which is in agreement with a previous study showing that A− was the predominant G6PDd allelic variant in some countries in Latin America and the Caribbean [2]. Furthermore, the frequency of the G6PD A genotypes found in this study was similar to a recent study performed in Honduras that detected 11.81 % of individuals with A− genotype and 4.27 % for A+ [12]. These similarities are probably due to the population structure evaluated in these two studies, mainly Afro-descendants located in coastal regions. In contrast, the frequency of G6PD A− genotype found in a study performed in Venezuela was lower (2.56 %) [13].

Tumaco (26.66 %) and Buenaventura (24.39 %) showed the highest allelic G6PDd frequencies (Fig. 3). The population of these two municipalities is mainly Afro-descendants (~90 %). Interestingly, these municipalities are malaria endemic sites in Colombia with a predominance of *P. vivax* malaria infections. The lowest proportion of individuals with G6PDd variants was found in Quibdo.

When the enzymatic activity was evaluated, a greater proportion of G6PD deficient females compared to deficient males was found. However, from genotyping analysis, the proportion of hemizygous males with G6PDd variants was higher than females (heterozygous or homozygous). In Honduras, a greater proportion of females with G6PDd allelic variants were reported compared to hemizygous males [12].

In some individuals it was founded G6PDd probably associated with the presence of A + and A− genotypes. A significant association between deficient enzyme activity and the presence of G6PDd genetic variants was found (p = 0.04098). Eight individuals presented functional G6PDd and displayed the corresponding genetic variants, however, these variants were not detected in the remaining 20 individuals that showed reduced G6PD enzyme activity. A plausible explanation is that G6PDd in these individuals is probably associated with other variants not evaluated in this study, which have not been characterized for the Colombian population and could be identified in the future by DNA sequencing of all exons.

This is the first report regarding frequency of G6PDd allelic variants in regions with endemic *P. vivax* malaria on the Colombian Pacific coast. Additionally, this study shows an important association between genotype and phenotype in these regions. Previous studies did not report allelic variants in individuals with reduced G6PD activity [22]. Here the A− genotype was detected in most of the samples with severe G6PDd (Table 3). It is surprising that no reports on haemolysis associated with the use of PQ use are available from these regions despite its frequent use and apparent good treatment compliance.

Used for treatment of both *P. falciparum* (the majority of malaria cases in Africa) and *P. vivax* (70 % of malaria cases in Colombia) PQ is important for elimination of liver hypnozoites in *P. vivax* malaria. The PQ regimen for *P. vivax* malaria consists of a much higher dose than the currently recommended WHO single low dose (0.25 mg base/kg) to block *P. falciparum* malaria transmission which is associated with a considerably lower risk of haemolytic toxicity [3]. Acute haemolytic anaemia (AHA) is the most common complication triggered by PQ in G6PD deficient-individuals in Latin America and the Caribbean region [2]. A recent report showed that G6PD A− was the allelic variant present in autopsy tissue samples from a previously reported death in the Brazilian Amazon due to PQ treatment of *P. vivax* malaria [33, 34]. Therefore, in Latin American countries such as Colombia and Brazil where *P. vivax* is the main parasite causing malaria, it is recommended that PQ administration requires prior determination of G6PD status (normal or deficient), However, this recommendation is not followed in most endemic countries of the continent, and it is uncertain what proportion of malaria cases, mainly *P. vivax* that are treated with therapeutic protocols including PQ, develop haemolysis. Substantial reduction of malaria by implementation of mass drug administration (MDA) using PQ has been observed in regions with high prevalence of *P. vivax* and varying prevalence of G6PDd [35]. The particularly high prevalence of sub-microscopic infections in Colombia [28] must be considered by malaria control programmes. The implementation of MDA through mass PQ prophylactic treatment (MPPT) [36], including sub-microscopic carriers, is possibly an important approach to reduction of transmission in Colombia. Such implementation would benefit from development of a suitable method for diagnosis of G6PDd in these locations. Testing for G6PDd is currently limited by cost, infrastructure, and logistics.

This study offered estimates of G6PDd prevalence in malaria endemic areas with considerable populations of African descent in a relatively small sample, a limitation of our study. Importantly, genotyping for the first time in these regions showed a high proportion of the G6PD A− variant in these populations. However, more information is needed about G6PDd prevalence in malaria endemic areas in Colombia, and other endemic countries of the Latin American region, including additional variants not evaluated in this study that may be associated with G6PDd in 20 individuals with no identified *G6PD* alleles. Further mapping of G6PDd prevalence and variants will help design appropriate malaria elimination strategies including MDA with PQ. A prospective study with a larger sample size including a close follow up to detect potential development of haemolysis and haemolytic anaemia in *P. vivax* patients under PQ treatment was recently initiated in collaboration with the MoH of Colombia. The MoH is currently preparing pilot activities towards malaria elimination which include the introduction of PQ treatment for *P. falciparum* cases, and more intensive search and treatment of asymptomatic cases, which are mostly *P. vivax* cases demanding 2 week PQ regimens.

## Conclusions

A high frequency of G6PD A− genotype, followed by A+ genotype was found in the analysed population, along with a moderately high proportion (6.56 %) of G6PDd based on enzymatic activity. Although 70 % of national malaria cases correspond to *P. vivax* infections and are treated for 14 days with PQ, no official reports of PQ-induced haemolytic crises are available. These results underscore the need to more carefully assess G6PDd in malaria endemic populations, certainly in a larger population, including other variants and sequencing as well as clinical follow up of PQ response. This study emphasizes the need to implement appropriate G6PDd diagnostic methods, particularly in view of possible MDA with PQ for both *P. vivax* and *P. falciparum* elimination.
