# Erratum to: Glucose-6-phosphate dehydrogenase deficiency prevalence and genetic variants in malaria endemic areas of Colombia

**DOI:** 10.1186/s12936-016-1383-6

**Published:** 2016-07-07

**Authors:** Sócrates Herrera Valencia, Iván Darío Ocampo, María Isabel Arce-Plata, Judith Recht, Myriam Arévalo-Herrera

**Affiliations:** Caucaseco Scientific Research Center/Malaria Vaccine and Drug Development Center, Carrera 37 2B No. 5 E‑08, Edificio de profesionales Bambú, Cali, Colombia; Centro Latino Americano de Investigación en Malaria (CLAIM), Cali, Colombia; Facultad de Salud, Universidad del Valle, Cali, Colombia

## Erratum to: Malar J (2016) 15:291 DOI 10.1186/s12936-016-1343-1

After publication of the original article [1], it was brought to the authors’ attention that there were errors in the final published version of Table 2. In the ‘Total’ row, the value in column 8 [Mean Hb (g/dL)] should have been ‘12.51’, not ‘23.00’; the value in column 10 [Max Hb (g/dl)] should have been ‘23.00’, not ‘23.80’. The original Table has been corrected, and is also published correctly in this erratum.

**Table 2:** Prevalence of G6PDd and summary statistics for G6PD enzyme activity and haemoglobin values for gender classification and areas of study

	n	Min		Median		Mean		Max		Deficiency n (%)	Confidence intervals
		G6PD (U/grHb)	Hb (g/dL)	G6PD (U/grHb)	Hb (g/dL)	G6PD (U/grHb)	Hb (g/dL)	G6PD (U/grHb)	Hb (g/dL)		
Gender											
Male	175	1.40	7.10	7.51	12.90	7.72	12.99	23.8	23.00	7 (4.00)	[1.76,8.39]
Female	251	1.93	7.10	7.94	12.00	7.79	12.18	15.8	19.70	21 (8.37)	[5.38,12.68]
Area											
Buenaventura	118	1.70	7.10	7.38	12.00	7.34	12.26	23.80	19.70	15 (12.71)	[7.53,20.41]
Tierralta	119	2.61	9.00	8.42	12.40	8.50	12.67	15.80	23.00	4 (3.36)	[1.08,8.90]
Tumaco	89	1.40	7.40	8.30	12.70	8.04	12.51	15.50	18.00	7 (7.87)	[3.49,16.05]
Quibdo	100	4.17	9.00	6.64	12.20	7.11	12.61	12.65	17.70	2 (2.00)	[0.35,7.74]
Total	426	1.40	7.10	7.76	12.30	7.76	12.51	23.80	23.00	28 (6.56)	[4.49,9.47]

In addition, the authors also noticed minor typos in the Abstract, the “Results” and the “Discussion” sections. The original article has been updated to rectify these errors.
